# Benzochromanone and Benzochromene Natural Products: Synthetic Strategies and Total Syntheses

**DOI:** 10.1002/tcr.202500265

**Published:** 2025-12-05

**Authors:** Takuya Kumamoto

**Affiliations:** ^1^ Department of Synthetic Organic Chemistry Graduate School of Biomedical and Health Sciences Hiroshima University Hiroshima Japan

**Keywords:** benzochromenes, Diels‐Alder reaction, spiro compounds, total synthesis, xanthones

## Abstract

This account overviews our synthetic strategies for natural products featuring benzochromanone and benzochromene frameworks. The total synthesis of monomeric benzochromanones, particularly xanthones and benzochromanones is achieved. Key accomplishments include the development of a versatile synthetic approach for constructing xanthone frameworks via spirochromanone intermediates and the successful total syntheses of (±)‐4‐deoxyblennolide C, (+)‐blennolide C, and chromanone lactone gonytolide C. The asymmetric total synthesis of benzochromene (*R*)‐(+)‐teretifolione B and the first asymmetric synthesis of (*R*)‐(+)‐methylteretifolione B are also achieved. The Diels–Alder reaction between benzyne derived from chromene precursors and oxygenated furans enabled efficient access to benzochromene derivatives. The enantioselective synthesis of teretifolione B and related compound was accomplished through the enzymatic resolution of racemic chromenes, and the reaction conditions were investigated to improve regioselectivity in key steps. These synthetic routes provide access to a diverse array of benzochromanone and benzochromene derivatives with potential biological activity.

## Graphical abstract

This work outlines synthetic strategies for natural products with benzochromanone and benzochromene cores. Key achievements include total syntheses of xanthones, blennolide C, gonytolide C, and teretifolione B. Spirochromanone intermediates, benzyne–furan Diels–Alder reactions, and enzymatic resolution enabled efficient and enantioselective access to diverse bioactive derivatives.

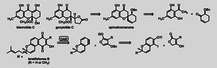

## 1 Introduction

**FIGURE 1:**
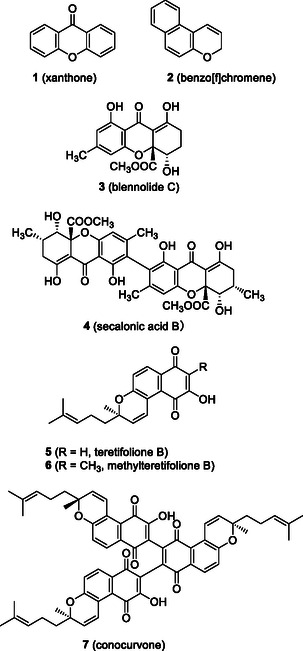
Representative benzochromanone and benzochromene natural products.

Natural products containing benzochromanone and benzochromene structures have been isolated from a wide range of natural sources, including plants, actinomycetes, lichens, and endophytes, and are found in both monomeric and oligomeric forms (Figure 1) [1, 2]. Our research focuses on the synthetic study of natural products that incorporate benzene‐fuzed chromanone (especially xanthones, (**1**) and chromene (**2**) units in their structures. Among them, partially hydrogenated xanthone blennolide C (**3**) was isolated from the culture broth of *Blennoria* sp., an endophytic fungus associated with *Carpobrotus edulis*, along with dimeric xanthone secalonic acid B (**4**) [3]. Teretifolione B (**5**) and its methyl derivative **6**, monomeric benzochromenes, were isolated from the Australian plant *Conospermum teretifolium*, along with conocurvone (**7**), a trimeric compound with anti‐HIV properties [4, 5]. In this account, we present our efforts toward the total synthesis of monomeric benzochromanones, including xanthones, its related chromanone lactones (CLs), and benzochromenes [6, 7, 8].

## 2 Synthetic Study of Xanthone Blennolide C and Chromanone Lactone Gonytolide C

**FIGURE 2:**
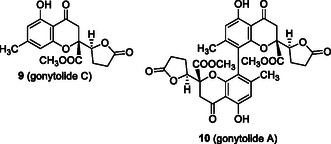
Structure of gonytolides C (**9**) and A (**10**).

**SCHEME 1:**
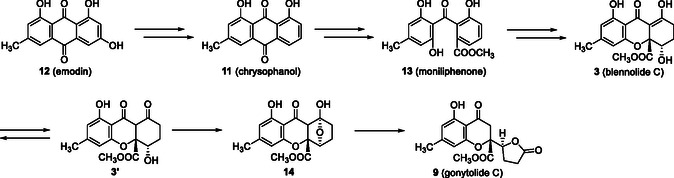
Proposed biosynthesis of blennolide C (**3**) and gonytolide C (**9**).

Xanthones (**1**) (a type of benzochromanone) are a class of natural substances found in plants, actinomycetes, fungi, and lichens. A particular subset, characterized by the partial hydrogenation or oxygenation of one of the benzene rings, including those with dimeric structures, has attracted attention because of its unique structural characteristics and biological activities. Xanthone dimers, referred to secalonic acid B (**4**) [9], are metabolites of *Claviceps purpurea* and the series of the secalonic acids are noted for their biological properties, such as anticancer [10] and antimicrobial activity [11]. The monomeric units of these dimers were identified as the blennolide class by Zhang et al. [3] Additionally, chromanone lactones (CLs) have been reported to be related to these xanthones. For instance, Kikuchi et al*.* [12] reported the isolation of gonytolide C (**9**) and the corresponding dimer gonytolide A (**10**) (Figure 2) from the fungus *Gonytrichum* sp., the latter of which exhibited innate immunosuppressive properties. The biosynthetic pathway of blennolide C (**3**) and gonytolide C (**9**) has been proposed as part of neosartorin biosynthesis (Scheme 1) [13, 14]. Chrysophanol (**11**), originating from emodin (**12**), undergoes Baeyer‐Villiger oxidation and ring‐opening, followed by esterification to form moniliphenone (**13**), which is converted to blennolide C (**3**) through aromatic ring epoxidation, xanthone ring formation, and reduction. Moreover, gonytolide C (**9**) is thought to be derived from keto‐form **3′** of blennolide C (**3**) to 1,4‐epoxide **14**, and then, through a retro‐Dieckmann reaction [14, 15], transformed into gonytolide C (**9**).

**SCHEME 2:**
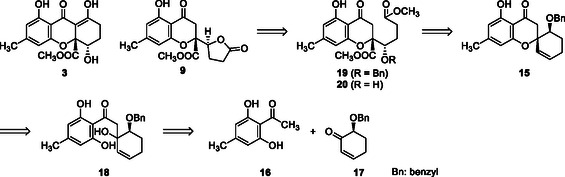
Retrosynthetic analysis of blennolide C (**3**) and gonytolide C (**9**).

Previously, the total synthesis of related xanthones and CLs has been reported [15, 16, 17, 18, 19, 20, 21, 22, 23, 24, 25, 26, 27, 28, 29, 30, 31]. Among them, asymmetric total synthesis of gonytolide C (**9**) was reported [23, 24], however total synthesis of blennolide C (**3**) was achieved only the racemic forms [23]. For example, Sudhakar et al*.* reported the total synthesis of racemic blennolide C (**3**) and optically active gonytolide C (**9**) through the Aldol reaction between acetophenone derivatives and butyrolactone containing α‐keto ester followed by diastereoselective intramolecular cyclization as key steps [23]. Our strategy was designed not only for the total synthesis of blennolides but also for their derivatives with varying ring sizes. The construction of asymmetric quaternary stereocenters is a significant challenge in the synthesis of natural xanthone derivatives. Spiro compounds, a class of natural products and synthetic intermediates, feature quaternary carbon centers [32, 33]. Blennolide C (**3**) and gonytolide C (**9**) with an asymmetric quaternary carbon center can be synthesized via spirochromanone intermediates **15**. The synthesis of the latter involves the Aldol reaction of modified acetophenones **16** with α‐oxygenated cyclohexenone **17** to form adducts **18**, followed by cyclization. The xanthone framework is constructed through the oxidative cleavage of the alkene moiety in spirochromanone **15** and Dieckmann condensation of the chromanone and ester components in the side chain of chromanone ester **19**. The use of cycloalkenones other than 6‐membered rings yields derivatives with different ring sizes. Lactone formation from alcohol **20** (R = OH) can lead to CL gonytolide C (**9**) (Scheme 2).

### 2.1 Synthetic Studies Toward Blennolide C and Gonytolide C

**SCHEME 3:**
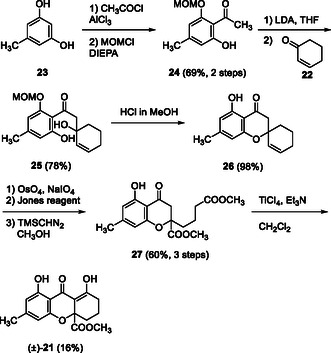
Total synthesis of (±)‐4‐deoxyblennolide C ((±)‐**21**).

We commenced our synthetic study of (±)‐4‐deoxy derivative **21** of blennolide C (**3**), using simple cyclohexenone (**22**). Friedel–Crafts acylation of 5‐methylresorcinol (**23**) with acetyl chloride using AlCl_3_ followed by mono protection as MOM ether gave hydroxyacetophenone **24**. The Aldol reaction of **24** and cyclohexenone (**22**) using two equivalents of LDA afforded Aldol adduct **25**, which was treated with HCl in methanol to yield spirochromanone **26**. Subsequent Lemieux‐Johnson oxidation of the cyclohexene moiety and Jones oxidation of the corresponding dial, followed by ester formation yielded diester **27**. This compound was treated with TiCl_4_ in the presence of Et_3_N [19] to afford the desired racemic or (±)‐4‐deoxyblennolide ((±)‐**21**) along with its corresponding methyl enol ether (Scheme 3) [34].

**SCHEME 4:**
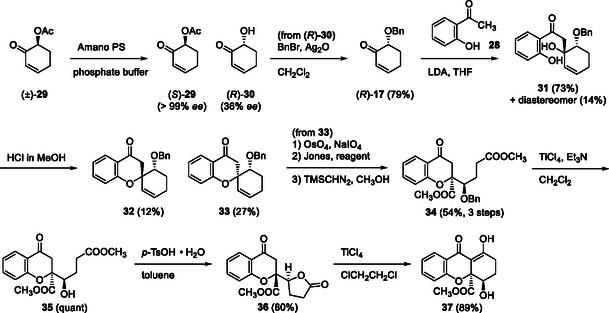
Total synthesis of demethylated blennolide C (**37**) and gonytolide C (**36**).

Next, we examined the synthesis using optically active α‐oxygenated cyclohexenone **17** and simple *o*‐hydroxyacetophenone (**28**). Racemic acetate (±)‐**29** was subjected to enzymatic kinetic resolution [35] to afford optically active acetate (*S*)‐**29** with the desired absolute configuration and high optical purity (>99%ee). However, trials for the hydrolysis of acetate **29** were not successful under the acidic and basic conditions we examined; therefore, alcohol (*R*)‐**30** with a lower ee (36% ee) generated by enzymatic resolution was used for further experiments. The application of benzyloxycyclohexenone (*R*)‐**17** derived from alcohol (*R*)‐**30** was subjected to an Aldol reaction with acetophenone **28**, affording Aldol adduct **31** as a mixture of diastereoisomers. This was further treated with HCl in methanol to afford spirochromanones **32** and **33** as a mixture of diastereomers. The separated spirochromanone **33** with the desired relative configuration was subjected to oxidative cleavage of the alkene part followed by esterification to afford diester **34**. Treatment of **34** with the TiCl_4_/Et_3_N system afforded deprotected alcohol **35**, which was treated with *p*‐TsOH to afford CL **36**, the model CL of gonytolide C (**9**). Dieckmann cyclization of **35** using TiCl_4_ in refluxed 1,2‐dichloroethane afforded demethylated blennolide C (**37**) (Scheme 4).

**SCHEME 5:**
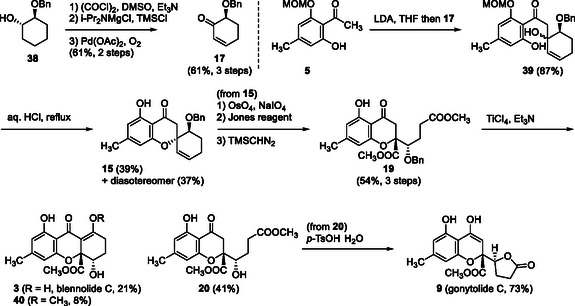
Total synthesis of blennolide C (**3**) and gonytolide C (**9**) via spirochromanone **15**.

To achieve the total synthesis of natural products, synthetic procedure of optically active cyclohexenone **17** was redesigned, using the enzymatic resolution of racemic benzyloxycyclohexanol (±)‐**38** [36], followed by Swern oxidation and Larock‐type dehydrogenation [37]. A similar procedure for the construction of diester **19** was adopted using acetophenone **5** and cyclohexenone **17** with (*S*) configuration. Treatment of diester **19** with TiCl_4_/Et_3_N afforded (+)‐blennolide C (**3**), its methyl enol ether **40**, and deprotected alcohol **20**. The latter **20** was then converted to gonytolide C (**9**) using *p*‐TsOH in refluxed toluene (Scheme 5). As a result, the first total synthesis of optically active blennolide C (**3**) was achieved [38].

## 3 Synthetic Study for Benzochromene Teretifoliones

**FIGURE 3:**
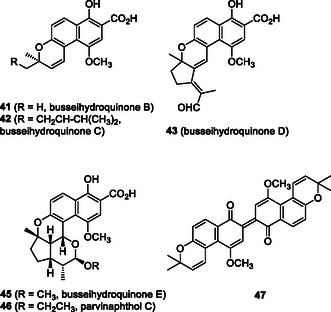
Representative benzochromene natural products **41–47**.

Benzochromenes (pyranonaphthalenes) have been isolated from various plants (Figure 3) [39]. Among them, busseihydroquinones B‐D (**41–43**) and pyranonaphthalene **44**, isolated from *Pentas bussei*, were reported as antiplasmodial compounds [40]. Pentacyclic busseihydroquinone E (**45**), the corresponding ethoxy derivative parvinaphthol C (**46**), and dimeric benzochromene **47** were isolated from *P. parvifolia* [40]. Several monomeric benzochromenes, including teretifolione B (**5**) and its methyl derivative **6** were isolated from *Conospermum teretifolium* [40]. Further research on the activity‐guided isolation of the same plant resulted in the isolation of a trimeric analog, conocurvone (**7**)*.* Notably, the trimeric conocurvone (**7**) demonstrated anti‐HIV‐1 activity; however, the monomeric teretifolione B shows no such activity [4, 5]. Several groups have been involved in the synthesis of conocurvone (**7**) and related monomeric species. The semisynthesis of conocurvone (**7**) from isolated teretifolione B (**5**) was reported by Boyd et al*.* [4, 5] to elucidate the structural correlation. The enantioselective total synthesis of teretifolione B (**5**) was reported by Vander Velde and Jacobsen through the kinetic resolution of racemic chromenes [41]. Methylteretifolione B (**6**) was synthesized in racemic form by Stagliano et al., via regioselective directed ortho metalation for the introduction of methylgroup [42].

**SCHEME 6:**
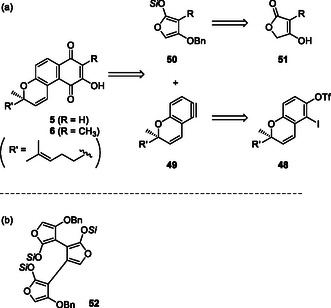
(a) Retrosynthesis of teretifoliones B (**5**, **6**). (b) Structure of trimeric furan as a presumed diene for DAR.

Our synthetic study of teretifolione B (**5**) is based on the preparation of optically active benzochromene **48** and the Diels–Alder reaction (DAR) of pyranobenzyne **49** [43, 44, 45] and oxygenated furans **50**, which are converted from tetronic acids **51** (Scheme 6a). In the future, trimeric furan **52** (Scheme 6b) could be adopted in DAR with three equivalents of benzyne **49** for the synthesis of trimeric benzochromene conocurvone (**7**). Here, we provide an overview of the synthetic studies on teretifoliones **5** and **6**. We first explored DAR using methylated furan **50** (R = CH_3_) for the synthesis of methylteretifolione B (**6**) because the asymmetric total synthesis of **6** had not been reported at that time.

### 3.1 Synthesis of Model Naphthoquinones via Diels–Alder Reactions of Benzynes and Furans

**SCHEME 7:**
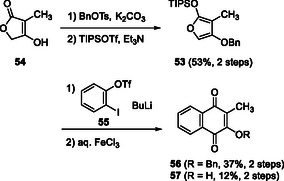
Synthesis of naphthoquinones **56** and **57** via DAR of benzyne and furan **53**.

We examined the synthesis of siloxyfuran **53** (R = CH_3_) and DAR using simple benzyne. *O*‐Selective benzylation of methyltetronic acid (**54**) was achieved using BnOTs, and the resulting tetronate was converted to siloxyfuran **53**. Treatment of siloxyfuran **53** with benzyne generated from triflate **55** and *n*‐BuLi afforded benzylated hydroxynaphthoquinone, which was treated with FeCl_3_ to convert to naphthoquinone **56** and naphthol pthiocol (**57**) (Scheme 7) [46].

**SCHEME 8:**
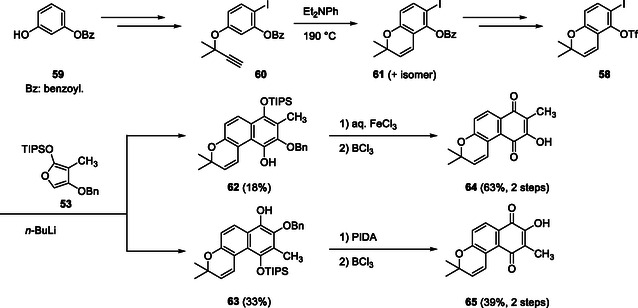
Synthesis of naphthoquinones **64** and **65** via DAR of benzyne precursor **58** and furan **53**.

Chromene‐derived benzyne precursor **58** with 2,2‐dimethyl groups was prepared as follows: commercially available resorcinol monobenzoate (**59**) was converted to iodinated propargyl ether **60**. The Claisen rearrangement of **60** afforded chromene **61**, and the subsequent ester exchange yielded iodo triflate **58** as a benzyne precursor. Treatment of **58** with *n*‐BuLi in the presence of furan **53** afforded two regioisomeric DA adducts **62** and **63**, in which the desired regioisomer **62** was obtained as a minor component. Both adducts were treated with FeCl_3_ or PIDA, followed by debenzylation using BCl_3_ to afford chromenoquinone **64** with the desired regiochemistry and its regioisomer **65** (Scheme 8) [47].

### 3.2 Total Synthesis of Teretifoliones

**SCHEME 9:**
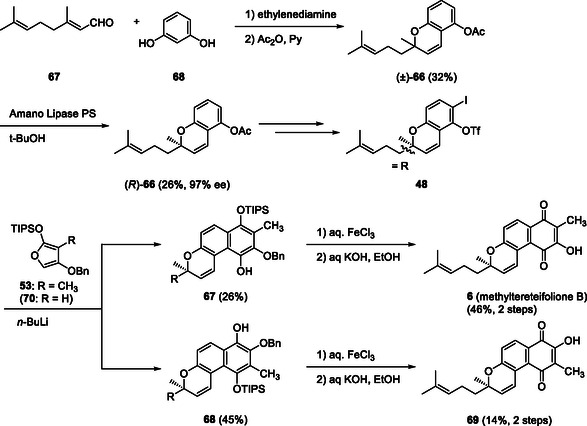
Synthesis of methylteretifolione B (**6**) and its regioisomer **69** via DAR of benzyne precursor **48** and furan **53**.

Next, we examined the synthesis of optically active methylteretifolione B (**6**). Optically active chromene acetate **66** with a 4‐methypent‐3‐enyl substituent was prepared by the condensation of citral (**67**) and resorcinol (**68**) in the presence of ethylenediamine using a modified reported procedure [48], followed by the enzymatic resolution of racemic acetate (±)‐**66** using Amano PS in the presence of *t*‐BuOH [49]. The application of hexanoate or chloroacetate instead of acetate in **66** afforded the ester with a lower ee [50]. After the conversion of the acetate to iodo triflate **48** in a similar manner, *n*‐BuLi‐mediated DAR with siloxyfuran **53** was performed, affording regioisomeric Diels–Alder adducts **67** and **68** in 26% and 45% yields, respectively. Both regioisomers were subjected to hydroquinone oxidation and benzyl ester hydrolysis to afford methylteretifolione B (**6**) and its regioisomer **69**. This result was the first example of total synthesis of optically active methylteretifolione B (**6**) (Scheme 9). In the course using siloxyfuran **70** without a methyl group, the total synthesis of teretifolione B (**5**) was also achieved [51].

### 3.3 Solvent and Temperature Effects on the Regioselectivity of DAR

**FIGURE 4:**
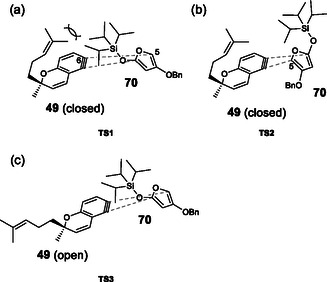
Plausible transition state models on DAR with benzyne **49** and furan **70**. (a) The model to afford undesired cycloadduct **72** with benzyne **49** in the “closed” confirmation. (b) The model to afford desired cycloadduct **71** with **49** in the “closed” confirmation. (c) The model to afford undesired cycloadduct **72** with benzyne **49** in the “open” confirmation.

The effects of the solvent and temperature on the regioselectivity of DAR were examined using racemic iodo triflate (±)‐**48** and siloxyfuran **70** (Table 1). The reaction under the original conditions (in THF, −78°C) afforded desired benzochromene **71** and undesired **72** in a 1:1.7 ratio, in which the undesired isomer was preferential (run 1). The regioselectivity was independent of temperature (runs 1–3). In the reaction in various solvents, DME gave a result similar to that of THF (run 4); however, the reactions in less polar solvents, such as hexane and toluene, resulted in an increase in the ratio of undesired **72** (runs 5 and 6). We speculate that the regioselectivity is primarily governed by the electrostatic interactions between the electron‐rich 5‐position of furan **70** and the electrophilic 6‐position of benzyne **49**, favoring the formation of undesired isomer **72**. Although steric repulsion exists between the side chain of benzyne and the bulky TIPS group in furan, it is overridden by the electrostatic effect (**TS1** > **TS2**) (Figure 4a,b). In less polar solvents, a conformational change in the benzyne side chain from “closed” to “open” conformation reduces steric hindrance between furan **70** and the side chain in **49**, thus leading to further increase of the formation of **72** (**TS3** > **TS1**) (Figure 4a,c) [51].

**TABLE 1:** Effect of solvents and conditions on the regioselectivity of DAR with (±)‐**48** and **70**.

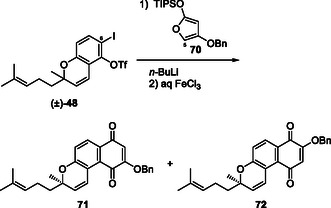			
Run	Solvent	Conditions	**71** : **72**
1	THF	−78°C, 20 min	1 : 1.7
2	THF	−40°C, 20 min	1 : 1.8
3	THF	0°C, 20 min	1 : 1.9
4	DME	−78°C, 30 min	1: 1.7
5	Hexane	−78°C, 30 min	1 : 3.3
6	Toluene	−78°C, 30 min	1 : 3.2

## 4 Summary and Outlook

In summary, this research has significantly advanced the total synthesis of benzochromanone and benzochromene natural products, exemplified by the successful construction of complex frameworks such as blennolides, gonytolide, and teretifoliones. The development of a versatile synthetic strategy utilizing spirochromanone intermediates, the achievement of total syntheses of key natural products, and the optimization of Diels–Alder and enantioselective processes have collectively broadened access to a diverse array of bioactive compounds. These accomplishments not only demonstrate the power and flexibility of the developed methodologies but also lay a strong foundation for future exploration.

## Conflicts of Interest

The author declares no conflicts of interest.

## Data Availability

Data sharing is not applicable to this article as no new data were created or analyzed in this study.

## References

[1] A. Gaspar , E. M. P. J. Garrido , F. Borges , and J. M. P. J. Garrido , “Biological and Medicinal Properties of Natural Chromones and Chromanones,” ACS Omega 9 (2024): 21706. 38799321 10.1021/acsomega.4c00771 PMC11112580

[2] Y. Xi , H. Wang , L. Sun , X. Ma , S. Zhang , and Z. Zhang , “Recent advances in the structures and bioactivities of benzopyrans derived from marine fungi: a review,” Frontiers in Pharmacology 15 (2024): 1482316. 39512833 10.3389/fphar.2024.1482316 PMC11540774

[3] W. Zhang , K. Krohn , U. Flörke , et al., “New Mono‐ and Dimeric Members of the Secalonic Acid Family: Blennolides A–G Isolated from the Fungus *Blennoria* sp.,” Chemistry–A European Journal 14 (2008): 4913. 18425741 10.1002/chem.200800035

[4] L. A. Decosterd , I. C. Parsons , K. R. Gustafson , et al., “HIV inhibitory natural products. 11. Structure, absolute stereochemistry, and synthesis of conocurvone, a potent, novel HIV‐inhibitory naphthoquinone trimer from a *Conospermum* sp.,” Journal of the American Chemical Society 115 (1993): 6673.

[5] J. R. Dai , L. A. Decosterd , K. R. Gustafson , J. H. Cardellina , G. N. Gray , and M. R. Boyd , “Novel Naphthoquinones from *Conospermum incurvum* ,” Journal of Natural Products 57 (1994): 1511. 7853001 10.1021/np50113a006

[6] Synthetic Studies Towards Teretifolione B and Conocurvone are Already Reviewed in References 7 and 8.

[7] K. Katakawa and T. Kumamoto , “Chemistry of Anti‐HIV Active Trimeric Benzochromene Conocurvone: Synthetic Studies towards Monomeric Teretifolione B and Related Compounds,” Journal of Synthetic Organic Chemistry Japan 76 (2018): 722

[8] K. Katakawa and T. Kumamoto , “Chemistry of Anti‐Hiv Active Trimeric Pyranonaphthoquinone Conocurvone: Synthetic Studies towards Monomeric Teretifolione B and Related Compounds,” Heterocycles 100 (2020): 177.

[9] B. Franck , E. M. Gottschalk , U. Ohnsorge , and G. Baumann , “The Structure of Secalonic Acids A and B,” Angewandte Chemie, International Edition in English 3 (1964): 441.

[10] R. Hong , “Secalonic acid D as a novel DNA topoisomerase I inhibitor from marine lichen‐derived fungus *Gliocladium* sp. T31,” Pharmaceutical Biology 49 (2011): 796. 21495809 10.3109/13880209.2010.548817

[11] S. Dominique , P. G. Alex , E. Y. Christiane , Y. Dodehe , and K. N. Adèle , “Diversity of Endophytic Fungi Isolated from the Bark of Ceiba pentandra (L.) Gaertn., (Bombacaceae) and Antibacterial Potential of Secalonic Acid A Produced by *Diaporthe searlei* EC 321,” Chemistry & Biodiversity 20 (2023): e202301010. 37814192 10.1002/cbdv.202301010

[12] H. Kikuchi , M. Isobe , M. Sekiya , et al., “Structures of the dimeric and monomeric chromanones, gonytolides A‐C, isolated from the fungus *Gonytrichum* sp. and their promoting activities of innate immune responses,” Organic Letters 13 (2011): 4624. 21827134 10.1021/ol2018449

[13] Y. Matsuda , C. H. Gotfredsen , and T. O. Larsen , “Genetic Characterization of Neosartorin Biosynthesis Provides Insight into Heterodimeric Natural Product Generation,” Organic Letters 20 (2018): 7197. 30394754 10.1021/acs.orglett.8b03123

[14] N. Tabata , H. Tomoda , K. Matsuzaki , and S. Omura , “Structure and biosynthesis of xanthoquinodins, anticoccidial antibiotics,” Journal of the American Chemical Society 115 (1993): 8558.

[15] D. K. Winter Reviews , D. L. Sloman , and J. A. Porco Jr, “Polycyclic xanthone natural products: structure, biological activity and chemical synthesis,” Natural Product Reports 30 (2013): 382. 23334431 10.1039/c3np20122h PMC3615431

[16] T. Wezeman , S. Bräse , and K.‐S. Masters , “Xanthone dimers: a compound family which is both common and privileged,” Natural Product Reports 32 (2015): 6. 25226564 10.1039/c4np00050a

[17] A. M. Dilmaç , T. Wezeman , R. M. Bär , and S. Bräse , “Occurrence, synthesis and applications of natural and designed [3.3.3]propellanes,” Natural Product Reports 37 (2020): 224. 31140489 10.1039/c8np00086g

[18] G. Valdomir and L. F. Tietze , “Chromanone Lactones: A Neglected Group of Natural Products – Isolation, Structure Elucidation, Bioactivity, and Synthesis,” European Journal of Organic (2022): e202200201.

[19] K. C. Nicolaou and A. Li , “Total Syntheses and Structural Revision of α- and β- Diversonolic Esters and Total Syntheses of Diversonol and Blennolide C,” Angewandte Chemie, International Edition 47 (2008): 6579. 18651685 10.1002/anie.200802632 PMC2790823

[20] T. Qin , R. P. Johnson , and J. A. Porco Jr, “Vinylogous Addition of Siloxyfurans to Benzopyryliums: A Concise Approach to the Tetrahydroxanthone Natural Products,” Journal of the American Chemical Society 133 (2011): 1714. 21265529 10.1021/ja110698n PMC3099260

[21] L. F. Tietze , L. Ma , J. R. Reiner , S. Jackenkroll , and S. Heidemann , “Enantioselective Total Synthesis of (−)‐Blennolide A,” Chemistry–A European Journal 19 (2013): 8610. 23649592 10.1002/chem.201300479

[22] A. C. Meister , A. Encinas , H. Sahin , et al., “Total Synthesis of Blennolide Mycotoxins: Design, Synthetic Routes and Completion,” European Journal of Organic Chemistry (2014): 4861.

[23] G. Sudhakar , S. Bayya , V. D. Kadam , and J. B.Nanubolu , “Total synthesis of gonytolides C and G, lachnone C, and formal synthesis of blennolide C and diversonol,” Organic & Biomolecular Chemistry 12 (2014): 5601. 24953777 10.1039/c4ob00950a

[24] F. F. Li , D. J. Atkinson , D. P. Furkert , and M. A. Brimble , “A Convergent Synthesis of Gonytolide C Using an Intramolecular Oxa‐Michael Addition,” European Journal of Organic Chemistry (2016): 1145.

[25] J. Cui , R. Oriez , S. Samanta , H. Noda , T. Watanabe , and M. Shibasaki , “Catalytic Asymmetric Vinylogous Conjugate Addition of Butenolide to 2‐Ester‐Substituted Chromones: Access to Chiral Chromanone Lactones via Trapping of a Copper(I) Enolate by Trimethyl Borate,” Organic Letters 25 (2023): 8367. 37962864 10.1021/acs.orglett.3c03503

[26] Y. Li , S. Xin , R. Weng , X. Liu , and X. Feng , “Asymmetric synthesis of chromanone lactones via vinylogous conjugate addition of butenolide to 2‐ester chromones,” Chemical Science 13 (2022): 8871. 35975160 10.1039/d2sc02541h PMC9350614

[27] For the Synthesis of Dimeric Xanthones: X. Wu , T. Iwata , A. Scharf , T. Qin , K. D. Reichl , and J. A. Porco Jr, “Asymmetric Synthesis of Gonytolide A: Strategic Use of an Aryl Halide Blocking Group for Oxidative Coupling,” Journal of the American Chemical Society 140 (2018): 5969. 29658717 10.1021/jacs.8b02535 PMC5943148

[28] T. Qin and J. A. Porco Jr, “Total Syntheses of Secalonic Acids A and D,” Angewandte Chemie, International Edition in English 53 (2014): 3107. 10.1002/anie.201311260 PMC4098722 24519991

[29] T. Qin , S. L. Skraba‐Joiner , Z. G. Khalil , R. P. Johnson , R. J. Capon , and J. A. Porco Jr, “Atropselective syntheses of (‐) and (+) rugulotrosin A utilizing point‐to‐axial chirality transfer,” Nature Chemistry 7 (2015): 234. 10.1038/nchem.2173 PMC4339264 25698333

[30] D. Ganapathy , J. R. Reiner , L. E. Löffler , et al., “Enantioselective Total Synthesis of Secalonic Acid E,” Chemistry–A European Journal 21 (2015): 16807. 26447631 10.1002/chem.201503593

[31] Z. Xiao , Y. Li , and S. Gao , “Total Synthesis and Structural Determination of the Dimeric Tetrahydroxanthone Ascherxanthone A,” Organic Letters 19 (2017): 1834. 28357853 10.1021/acs.orglett.7b00592

[32] R. Rios , “Enantioselective methodologies for the synthesis of spiro compounds,” Chemical Society Reviews 41 (2012): 1060. 21975423 10.1039/c1cs15156h

[33] B. R. Raju and A. K. Saikia , “Asymmetric Synthesis of Naturally Occuring Spiroketals,” Molecules 13 (2008): 1942. 18794795 10.3390/molecules13081942 PMC6245485

[34] T. Kumamoto , S. Hasegawa , K. Adachi , and K. Katakawa , “Total Synthesis of (±)‐4‐Deoxyblennolide C via Spirochromanone,” Heterocycles 103 (2021): 1064.

[35] F. Oğuzkaya , E. Şahin , and C. Tanyeli , “Stereoselective synthesis of optically active cyclitol precursors via a chemoenzymatic method,” Tetrahedron, Asymmetry 17 (2006): 3004.

[36] K. Yamato , R. A. Bartsch , G. A. Broker , R. D. Rogers , and M. L. Dietz , “Synthesis of chiral trans‐anti‐trans‐isomers of dicyclohexano‐18‐crown‐6 via an enzymatic reaction and the solid‐state structure of one enantiomer,” Tetrahedron Letters 43 (2002): 5805.

[37] R. C. Larock , T. R. Hightower , G. A. Kraus , P. Hahn , and D. Zheng , “A simple, effective, new, palladium‐catalyzed conversion of enol silanes to enones and enals,” Tetrahedron Letters 36 (1995): 2423.

[38] K. Adachi , S. Hasegawa , K. Katakawa , and T. Kumamoto , “Total synthesis of (+)‐blennolide C and (+)‐gonytolide C via spirochromanone,” Tetrahedron Letters 58 (2017): 4479.

[39] R. Pratap and V. J. Ram , “Natural and Synthetic Chromenes, Fused Chromenes, and Versatility of Dihydrobenzo[*h*]chromenes in Organic Synthesis,” Chemical Reviews 114 (2014): 10476. 25303539 10.1021/cr500075s

[40] J. F. Bukuru , T. N. Van , L. Van Puyvelde , S. G. Mathenge , F. P. Mudida , and N. De Kimpe , “A benzochromene from the roots of *Pentas bussei* ,” Journal of Natural Products 65 (2002): 783. 12027769 10.1021/np0101604

[41] S. L. Vander Velde and E. N. Jacobsen , “Kinetic Resolution of Racemic Chromenes via Asymmetric Epoxidation: Synthesis of (+)‐Teretifolione B,” The Journal of Organic Chemistry 60 (1995): 5380.

[42] K. W. Stagliono and H. C. Malinakova , “Regioselective directed ortho metalation of 3*H*‐naphto[2,1‐*b*]pyrans. Synthesis of methylteretifolione B,” Tetrahedron Letters 39 (1998): 4941.

[43] Examples of Apprication of Chromene Substrates as a Precursor of Arynes, See: Y. Nakamura , Y. Sakata , T. Hosoya , and S. Yoshida , “Synthesis of Functionalized Benzopyran/Coumarin‐Derived Aryne Precursors and Their Applications,” Organic Letters 22 (2020): 8505. 33048552 10.1021/acs.orglett.0c03106

[44] Y.‐Z. Xu , J.‐W. Tian , F. Sha , Q. Li , and X.‐Y. Wu , “Concise Synthesis of Chromene/Chromane‐Type Aryne Precursors and Their Applications,” The Journal of Organic Chemistry 86 (2021): 6765. 33852309 10.1021/acs.joc.1c00493

[45] Y. Xu , F. Sha , and X. Wu , “Design of a Functional Chromene‐Type Kobayashi Precursor: Gram‐Scale Total Synthesis of Natural Xanthones by Highly Regioselective Aryne Annulation,” Chemistry–A European Journal 27 (2021): 1066. 33000486 10.1002/chem.202003805

[46] K. Katakawa , D. Yonenaga , T. Terada , et al., “Selective Synthesis of Benzyl Enol Ethers of β‐Dicarbonyl Compounds in Basic Condition and the Application towards Synthesis of Naphthoquinones,” Heterocycles 88 (2014): 817.

[47] K. Katakawa , A. Sato , M. Iwasaki , T. Horikawa , and T. Kumamoto , “Synthesis of a Natural Chromenoquinone via the Diels–Alder Reaction of Pyranobenzyne and Furan,” Chemical and Pharmaceutical Bulletin 62 (2014): 820. 25087635 10.1248/cpb.c14-00228

[48] Y. R. Lee , J. H. Choi , and S. H. Yoon , “Efficient and general method for the synthesis of benzopyrans by ethylenediamine diacetate‐catalyzed reactions of resorcinols with α,β‐unsaturated aldehydes. One step synthesis of biologically active (±)‐confluentin and (±)‐daurichromenic acid,” Tetrahedron Letters 46 (2005): 7539.

[49] J. Y. Goujon , F. Zammattio , and B. Kirschleger , “Synthesis of various 2H‐benzopyran compounds and their kinetic resolution by asymmetric hydrolysis of their racemic acetates mediated by lipases,” Tetrahedron, Asymmetry 11 (2000): 2409.

[50] M. Kainuma , A. Yamada , K. Katakawa , and T. Kumamoto , “Preparation of Optically Active 2,2‐Disubstituted 5‐Hydroxychromenes by the Enzymatic Resolution of Racemic Esters,” Heterocycles 97 (2018): 604.

[51] K. Katakawa , M. Kainuma , K. Suzuki , S. Tanaka , and T. Kumamoto , “Asymmetric total syntheses of teretifolione B and methylteretifolione B via Diels‐Alder reaction of optically active pyranobenzyne and substituted furans,” Tetrahedron 73 (2017): 5063.

